# Omentectomy Versus Omental Preservation During Gastrectomy for Gastric Cancer: Systematic Review and Reconstructed Individual Patient Data Meta-Analysis

**DOI:** 10.3390/jcm15218495

**Published:** 2026-11-01

**Authors:** Alberto Aiolfi, Antonio Farro, Matteo Calì, Francesco Cammarata, Calin Popa, Gianluca Bonitta, Marta Cavalli, Giampiero Campanelli, Antonio Biondi, Luigi Bonavina, Davide Bona

**Affiliations:** 1 Division of General Surgery, I.R.C.C.S. Ospedale Galeazzi—Sant’Ambrogio, 20151 Milan, Italy; antonio.farro@unimi.it (A.F.);; 2 Regional Institute of Gastroenterology and Hepatology O. Fodor Cluj-Napoca, University of Medicine and Pharmacy Iuliu Hatieganu Cluj-Napoca, Croitorilor 19-21, 400162 Cluj-Napoca-Napoca, Romania; 3 Galeazzi-Sant’Ambrogio Hospital, University of Insubria, 20157 Milan, Italy; 4 G. Rodolico Hospital, Surgical Division, Department of General Surgery and Medical Surgical Specialties, University of Catania, 95131 Catania, Italy; abiondi@unict.it; 5 Department of Pharmacy, Health and Nutrition Sciences, Azienda Ospedaliera di Cosenza, Division of General and Foregut Surgery, University of Calabria, 87036 Cosenza, Italy; luigi.bonavina@unimi.it; 6 Department of Biomedical Science for Health, University of Milan, 20157 Milan, Italy

**Keywords:** gastric cancer, gastrectomy, omentectomy, overall survival, disease-free survival, reconstructed individual patient data meta-analysis

## Abstract

**Background/Objectives:** The oncological benefit of routine omentectomy (OmR) during curative gastrectomy for gastric cancer (GC) remains controversial. While omentectomy has traditionally been performed to reduce peritoneal dissemination, the greater omentum also possesses immunological functions that may protect against tumor implantation. This study evaluated the impact of OmR versus omental preservation (OmP) on long-term survival using reconstructed individual patient data (IPD) and restricted mean survival time difference (RMSTD) meta-analysis. **Methods**: A systematic review was conducted according to PRISMA 2020 guidelines. Comparative studies reporting Kaplan–Meier curves for overall survival (OS) and/or disease-free survival (DFS) after OmR versus OmP were included. IPD were reconstructed from published survival curves, and pooled RMSTD estimates were calculated using random-effects multivariate meta-analysis. Flexible hazard-based regression models assessed time-varying treatment effects. Secondary outcomes included postoperative morbidity and operative outcomes. **Results**: Fifteen studies involving 5325 patients were included, of whom 54% underwent OmR. At 60 months, no significant difference in OS was observed for OmR vs. OmP (−0.79 months, 95% CI −2.13 to 0.54). Similarly, DFS did not differ significantly (−2.12 months, 95% CI −4.59 to 0.35). Time-varying hazard analyses demonstrated no survival advantage for OmR throughout follow-up. OmR was associated with significantly higher intraoperative blood loss (MD 42.9), overall complications (RR 1.17), and longer hospital length of stay (MD 0.49). Retrieved lymph nodes, anastomotic leak, operative time, and 30-day mortality were comparable. **Conclusions:** In this study, routine omentectomy during gastrectomy for gastric cancer was not associated with improved OS or DFS compared with omental preservation. These findings question the need for routine omentectomy and suggest that omental preservation may represent a reasonable surgical strategy in selected patients, pending confirmation from prospective randomized trials.

## 1. Introduction

In patients with advanced gastric cancer (GC), contemporary management comprises gastrectomy with D2 lymphadenectomy, combined with perioperative chemotherapy [1,2,3,4,5]. Traditionally, removal of the greater omentum and omental bursa formed part of the Japanese concept of radical gastrectomy, with the rationale of reducing localized tumor implantation and subsequent peritoneal recurrence [6,7,8]. While bursectomy is no longer advised, the clinical utility of routine omentectomy remains uncertain [9]. Because peritoneal dissemination represents the most common pattern of recurrence after gastrectomy for GC [10], concerns persist among surgeons that omental preservation could increase the risk of peritoneal relapse or postoperative small-bowel obstruction. Conversely, accumulating evidence suggests that the omentum may exert anti-inflammatory and tumor-protective effects, thereby potentially contributing to an antitumor immune response against exfoliated and free intraperitoneal tumor cells [11]. In particular, this putative antitumoral function is thought to be mediated by milky spots, which are predominantly located within the omentum, mesentery, and abdominal wall and represent secondary lymphoid structures capable of initiating early antitumor immune activity [12,13]. Despite this theoretical basis, a definitive benefit of omentectomy (OmR) in patients with GC has not been established. Previous retrospective studies comparing OmR versus omental preservation (OmP) reported no significant differences in overall survival (OS) or disease-free survival (DFS) [14,15,16,17,18,19,20,21,22,23,24,25,26,27,28]. At present, European Society for Medical Oncology (ESMO) guidelines do not provide a specific recommendation regarding omentectomy [1], whereas Japanese guidelines continue to issue a weak recommendation for omentectomy in cT3–4 gastric cancer [3]. Currently, two randomized clinical trials are evaluating the non-inferiority of omental preservation; however, their results are not yet available [29,30].

The purpose of this study was to assess the influence of OmR versus OmP on long-term survival following gastrectomy for GC. We performed a restricted mean survival time difference (RMSTD) and reconstructed individual patient data (IPD) meta-analysis to overcome limitations found in prior meta-analyses that typically utilize hazard ratios (HRs) or odds ratios (ORs) [31,32,33]. This methodology provides an absolute and clinically interpretable measure of treatment effect and is particularly useful when the proportional hazards assumption underlying conventional hazard-ratio analyses is not satisfied.

## 2. Materials and Methods

A systematic review was conducted and reported according to the PRISMA 2020 guideline [34]. Ethical approval was not required. Multiple databases, including Scopus, Web of Science, PubMed, Cochrane Central Library and Google Scholar were utilized for the search process [35]. The initial search took place in December 2025, was repeated in March 2026, and updated on 1 June 2026. MeSH terms (Medical Subject Headings), such as “omentectomy”, “gastrectomy,” “survival,” “overall survival,” “disease-free survival,” and “cancer specific survival” were combined with “AND” (Appendix A). All titles were reviewed, relevant abstracts were extracted, and the reference lists of identified articles were independently assessed by three authors (A.F., A.A., and D.B.). The systematic review has been registered with the PROSPERO database under registration number CRD420261354658.

### 2.1. Eligibility Criteria

Inclusion criteria: (a) studies comparing OmR vs. OmP for the treatment of GC; (b) studies reporting long-term OS, DFS, or cancer specific survival (CSS) Kaplan–Meier curves; (c) in case of study overlap published by the same institution, study group, or dataset we included the larger sample size or the most recent study. Exclusion criteria: (a) studies without a comparative analysis of OmR versus OmP, (b) studies that reported none of the predefined primary outcomes.

### 2.2. Data Extraction

The data collected included information on authors, year of publication, country, trial acronym, number of patients, sex, age (years), body mass index (BMI) (kg/m^2^), American Society of Anesthesiologists (ASA) physical status, tumor characteristics, tumor location, surgical approach (open vs. minimally invasive), duration of follow-up (months), OS, DFS, and CSS. Two authors (A.F. and A.A.) independently gathered all data, which was reconciled at the conclusion of the evaluation process. A third author (D.B.) reviewed the database to address any discrepancies and ensure accuracy.

### 2.3. Outcomes of Interest and Definitions

The primary outcomes included 5-year OS and DFS. OS was defined as the duration from surgery to death from any cause, with surviving patients censored at the last follow-up. DFS and RFS were analyzed within the same time-to-event outcome framework, using the endpoints as reported by the original studies. As event definitions were not consistently available, no assumptions were made regarding unreported endpoint components. Patients without an event were censored at the last documented disease-free follow-up. Secondary outcomes encompassed postoperative complications, duration of surgery, bleeding, anastomotic leak, intra-abdominal abscess, intestinal obstruction and 30-day mortality. Individual patient survival data were derived from Kaplan–Meier curves. OmR was defined as complete/total resection of the greater omentum, whereas OmP included partial omentectomy or preservation of the distal greater omentum, according to the operative definition used in each original study (Supplementary Table S1).

### 2.4. Quality Assessment and Assessment of Certainty of Evidence

The risk of bias of the included non-randomized studies was independently assessed by two reviewers (AA and AF) using the Risk Of Bias In Non-randomized Studies of Interventions (ROBINS-I) tool [36]. Assessment was performed with reference to the oncological outcomes of overall survival and disease-free/relapse-free survival. ROBINS-I evaluates seven domains: bias due to confounding, selection of participants, classification of interventions, deviations from intended interventions, missing data, measurement of outcomes, and selection of the reported result. Particular attention was given to confounding by indication, including differences in tumor stage and burden, surgical approach, extent of lymphadenectomy, perioperative systemic treatment, treatment period, institutional practice, and surgeon-related treatment selection. Domain-level judgments were classified as low, moderate, serious, or critical risk of bias, and an overall risk-of-bias judgment was assigned for each study according to ROBINS-I guidance. Disagreements between reviewers were resolved by discussion and consensus, with involvement of a third reviewer when required. The certainty of evidence for the primary oncological outcomes was assessed using the GRADE approach and GRADEpro [37,38]. Certainty was evaluated across the domains of risk of bias, inconsistency, indirectness, imprecision, and publication bias.

### 2.5. Statistical Analysis

Patient-specific time-to-event data were reconstructed from published Kaplan–Meier curves using GetData Graph Digitizer software (version 2.26) (accessed on 30 April 2026) [39,40]. Reconstructed individual patient data (IPD) were used to estimate survival functions and treatment effects for overall survival (OS) and disease-free survival (DFS). Whenever available, published numbers at risk were incorporated into the reconstruction process. The accuracy of the reconstructed data was independently assessed by two authors (AA and AF) through graphical comparison of the reconstructed and originally published Kaplan–Meier curves and, whenever available, by comparing hazard ratios estimated from reconstructed IPD with those reported in the original studies. The reconstruction was subsequently reviewed and validated by a medical statistician (GB). Restricted mean survival time (RMST) was defined as the expected event-free survival time up to a prespecified time horizon (τ), corresponding to the area under the survival curve from time zero to τ [41]. Treatment effects were expressed as the restricted mean survival time difference (RMSTD), calculated as the RMST for omentectomy (OmR) minus the RMST for omental preservation (OmP). Accordingly, positive RMSTD values indicated longer restricted mean survival with OmR, whereas negative values favored OmP. RMSTD estimates were expressed in months and reported with corresponding 95% confidence intervals (CIs). Study-specific RMSTD estimates were pooled using a frequentist random-effects multivariate meta-analysis accounting for within-study covariance across time points [42]. The time horizon was set at 60 months, corresponding to the clinically relevant 5-year survival endpoint. Pooled RMSTD estimates were calculated separately for OS and DFS where reported. To assess whether the effect of surgical treatment varied over time, reconstructed IPD were additionally analyzed using flexible hazard-based regression models with a normally distributed study-specific random intercept [43]. The baseline hazard function was modeled using a degree-3 exponential B-spline without internal knots, selected according to the Akaike information criterion (AIC). Time-varying treatment effects were modeled through interaction terms between surgical treatment and the baseline hazard function. Evidence of time-varying treatment effects was assessed using likelihood-ratio tests. Hazard functions and time-varying treatment effects were obtained from marginal model predictions. For dichotomous outcomes, treatment effects were expressed as risk ratios (RRs) with 95% confidence intervals (CIs). For continuous outcomes measured on the same scale across studies, mean differences (MDs) with 95% CIs were calculated; standardized mean differences (SMDs) were used when outcomes were reported using different scales or measurement approaches. Time-to-event outcomes were summarized using survival probabilities and restricted mean survival time (RMST); between-group differences in RMST were expressed as restricted mean survival time differences (RMSTDs) with 95% CIs at prespecified time horizons.

Secondary outcomes were pooled using random-effects models with the DerSimonian–Laird estimator, and between-study heterogeneity was quantified using the I^2^ statistic. Propensity score-matched cohorts were preferentially used when available; otherwise, unmatched data were considered. Studies reporting zero events in both treatment groups were excluded from the corresponding analyses. Given the substantial heterogeneity observed for some perioperative outcomes, pooled estimates were interpreted cautiously. All statistical tests were two-sided, and *p* values < 0.05 were considered statistically significant. Effect estimates are presented with 95% CIs. Statistical analyses were performed using R software (version 3.2.2; R Foundation for Statistical Computing, Vienna, Austria) [44].

## 3. Results

### 3.1. Literature Search

**Figure 1:**
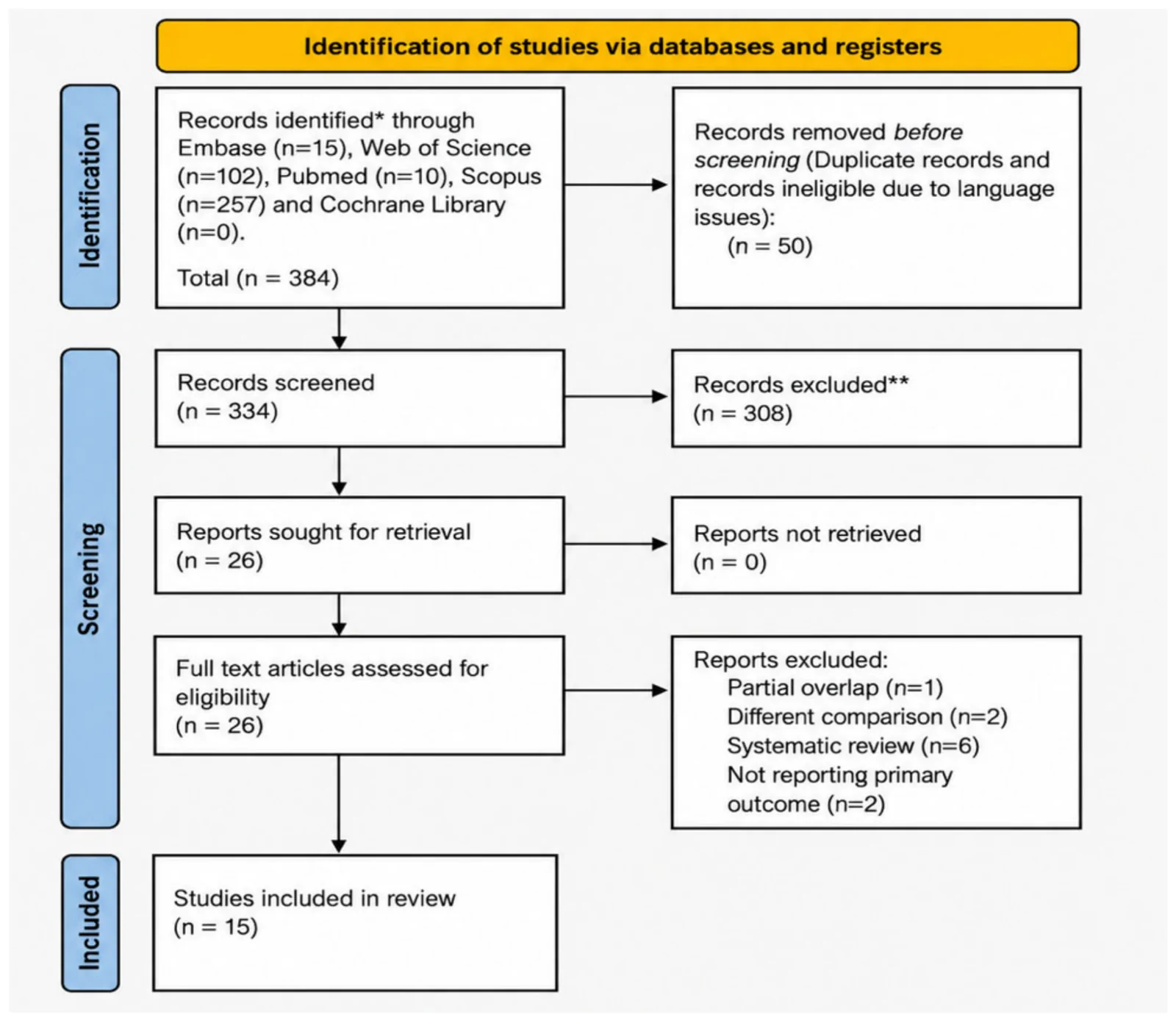
The Preferred Reporting Items for Systematic Reviews checklist (PRISMA) diagram. * Records excluded: conference abstracts, book chapters, conference posters, letters, editorials or unrelated topics. ** Records excluded: reviews, duplicate studies/same group research center; data not retrievable/subgroup not distinguishable, inclusion/exclusion criteria not met.

The initial search strategy identified 384 records. Following removal of duplicates, 334 records were screened, of which 308 were excluded after title and abstract assessment according to the predefined eligibility criteria. Additional exclusions comprised studies evaluating different interventions or comparisons (n = 2), review articles (n = 6), and studies not reporting the predefined primary outcomes (n = 2), and partial overlap (n = 1). Ultimately, 15 studies were included in the qualitative synthesis (Figure 1).

### 3.2. Risk of Bias Assessment

According to ROBINS-I, six studies were judged to have an overall moderate risk of bias and nine a serious risk of bias; no study was considered to be at low or critical risk of bias. The most frequent source of bias was residual confounding related to the non-random allocation of omentectomy or omental preservation. In several studies, treatment allocation was influenced by tumor characteristics, surgical approach, institutional practice, treatment period, or surgeon preference, and these factors were not always fully accounted for despite the use of propensity-score matching or multivariable adjustment. Additional concerns included incomplete control of prognostic covariates, differential or incomplete follow-up, exclusion of patients with missing data, and, in some studies, the close correlation between omental management and operative approach. Overall risk-of-bias judgments are summarized in Supplementary Table S2.

### 3.3. Systematic Review

The studies were published between 2009 and 2026 and originated from Japan (n = 4), South Korea (n = 5), Europe (n = 2), China (n = 2), Turkey (n = 1), and the United States (n = 1). Most studies were retrospective cohort investigations, frequently incorporating propensity score matching to mitigate selection bias. Overall, the included studies comprised 5325 patients with gastric cancer, of whom 2876 (54%) underwent gastrectomy with OmR (Table 1). Across studies, patient age ranged from 37 to 91 years, and men represented the majority of the study population (62%). ASA physical status was reported in nine studies, whereas preoperative BMI was available in ten studies. Tumor histology was described in ten studies, all of which reported adenocarcinoma; only one study provided data stratified by diffuse and intestinal histological subtypes. Distal and total gastrectomy accounted for 61% and 39% of procedures, respectively, largely according to tumor location and histology. Open and laparoscopic approaches were reported in 62.8% and 37.2% of cases, respectively. The extent of lymphadenectomy was heterogeneous and varied according to the operating surgeon, including D1, D1+, D2, or extended D2 dissections. Pathological tumor stage was defined in 8 studies: stage I (14%), stage II (32%), stage III (48.3%), and stage IV (5.7%). Neoadjuvant treatment was reported in four studies, perioperative FLOT chemotherapy in one study, and adjuvant treatment according to institutional protocols and contemporary guidelines in eight studies.

### 3.4. Reconstructed IPD Meta-Analysis

**Figure 2:**
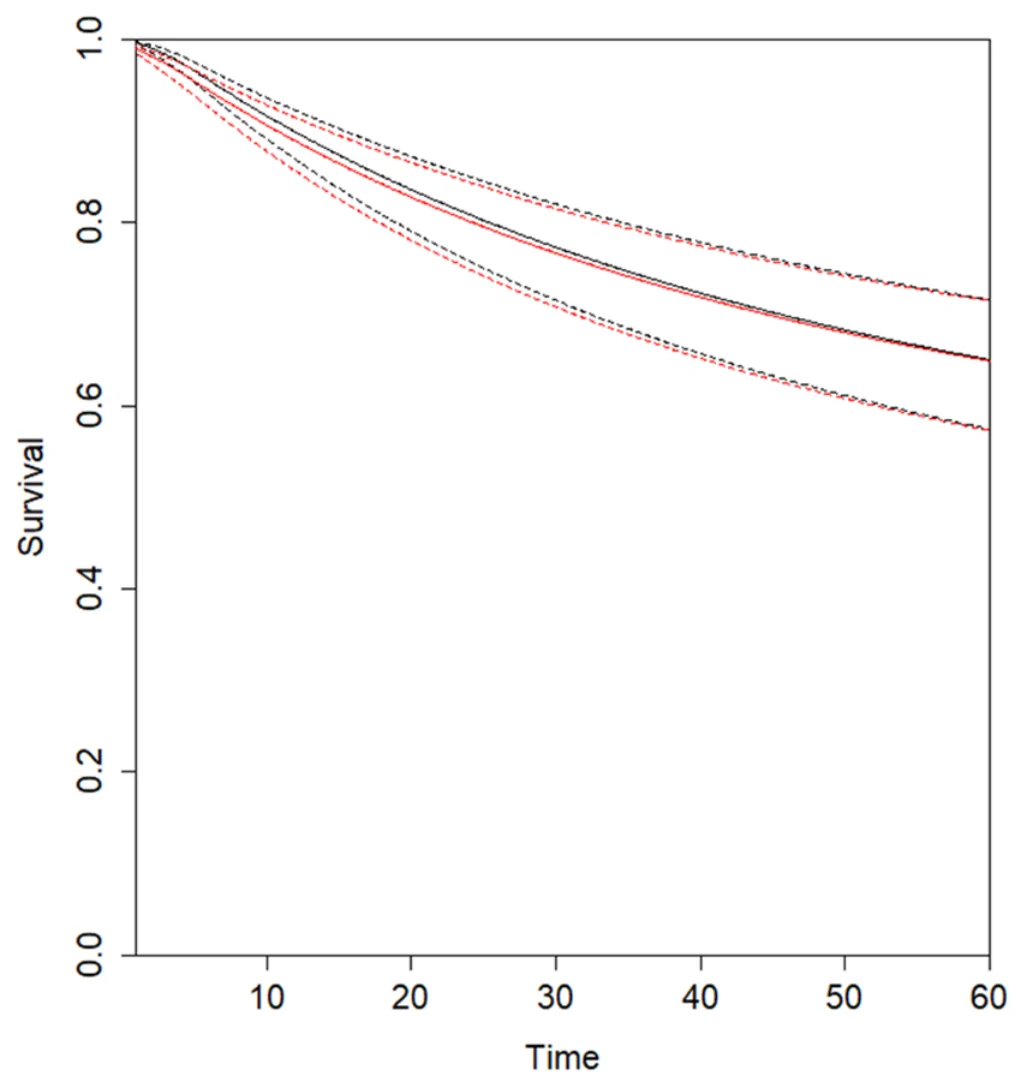
Estimated pooled overall survival (Y axis) for OmR (red line) and OmP (black line). Time (X axis) is expressed in months. The solid line represents the predicted overall survival curve, whereas the dotted lines denote the corresponding 95% confidence intervals.

**Figure 3:**
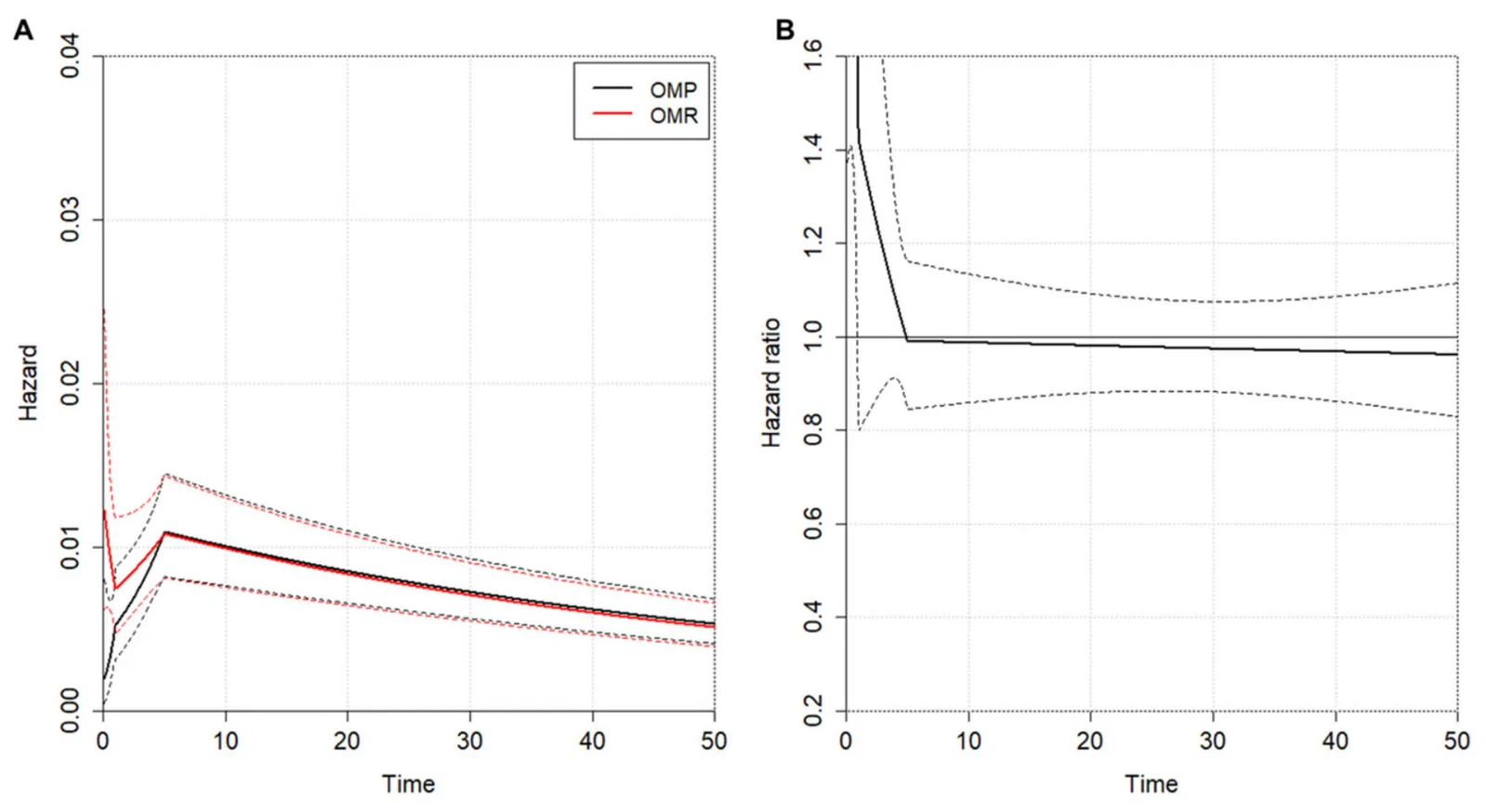
(**A**) Estimated time-dependent OS hazard functions for OmR (red line) and OmP (black line). (**B**) Estimated time-varying hazard ratio for OmR versus OmP. The solid line represents the predicted overall survival curve, whereas the dotted lines denote the corresponding 95% confidence intervals.

The clinical appraisal of the OS RMSTD was based on studies reporting Kaplan–Meier curves (n = 14). The RMSTD and the time horizons are detailed in Table 2. At the 60-month time horizon, the pooled RMSTD for OS (OmR minus OmP) was −0.79 months (95% CI, −2.13 to 0.54; *p* = 0.24), indicating no statistically significant difference in restricted mean overall survival between the two surgical strategies. For OS, sensitivity analyses restricted to propensity score-matched cohorts (n = 7 studies; RMSTD −0.65 months, 95% CI −2.45 to 1.05; *p* = 0.46) and studies at moderate risk of bias (n = 5 studies; RMSTD −0.43 months, 95% CI −1.98 to 1.66; *p* = 0.64) yielded results consistent with the primary analysis. The estimated pooled 5-year survival estimates were 65.2% for OmR and 65.3% for OmP (Figure 2). The likelihood-ratio test indicated evidence of a time-varying treatment effect (*p* < 0.001); however, the estimated time-varying hazard ratio did not show a statistically significant difference between OmR and OmP at any point up to 50 months of follow-up (Figure 3A,B).

**Figure 4:**
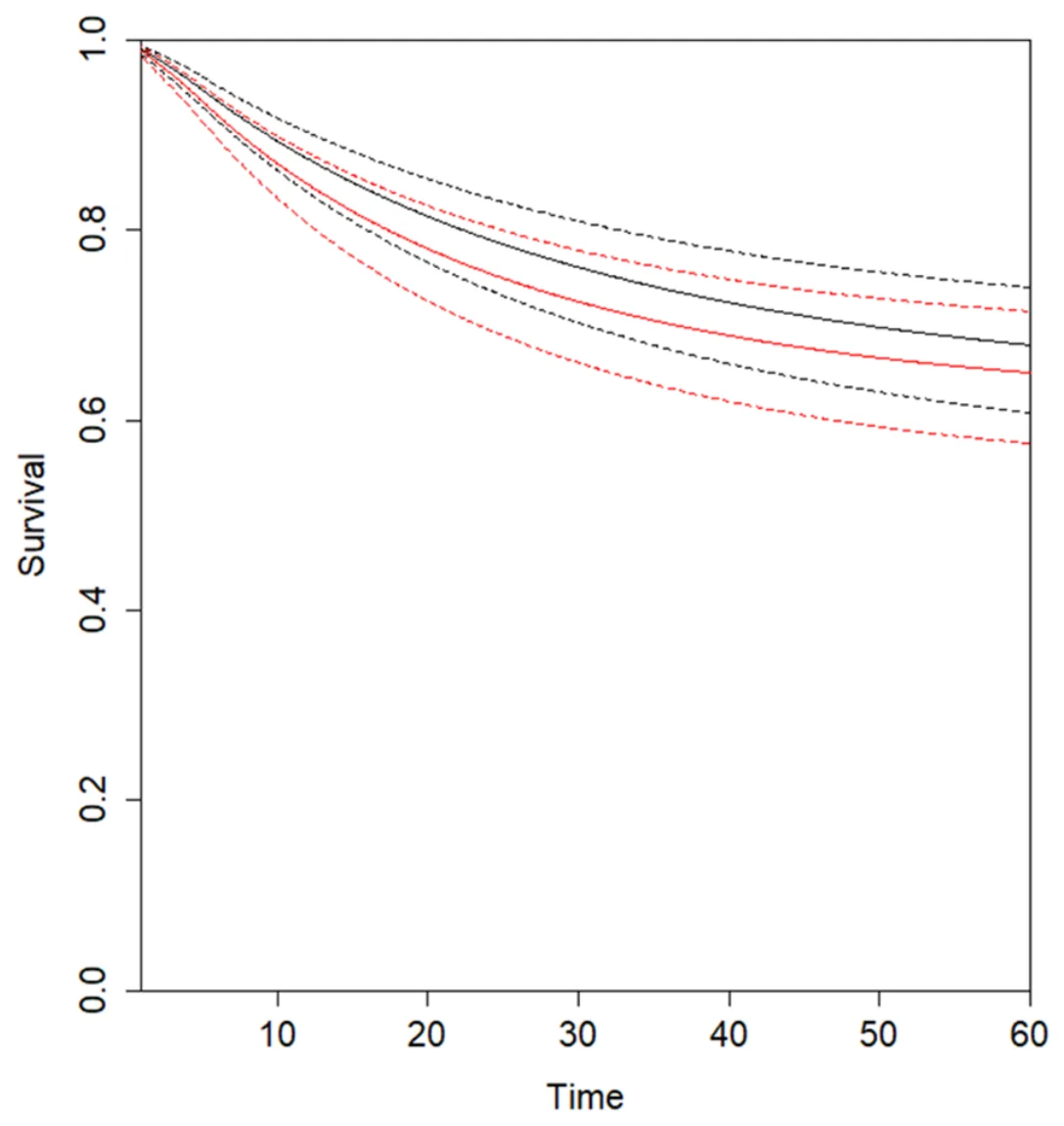
Estimated pooled disease-free survival (Y axis) for OmR (red line) and OmP (black line). Time (X axis) is expressed in months. The solid line represents the predicted disease-free survival curve, whereas the dotted lines denote the corresponding 95% confidence intervals.

**Figure 5:**
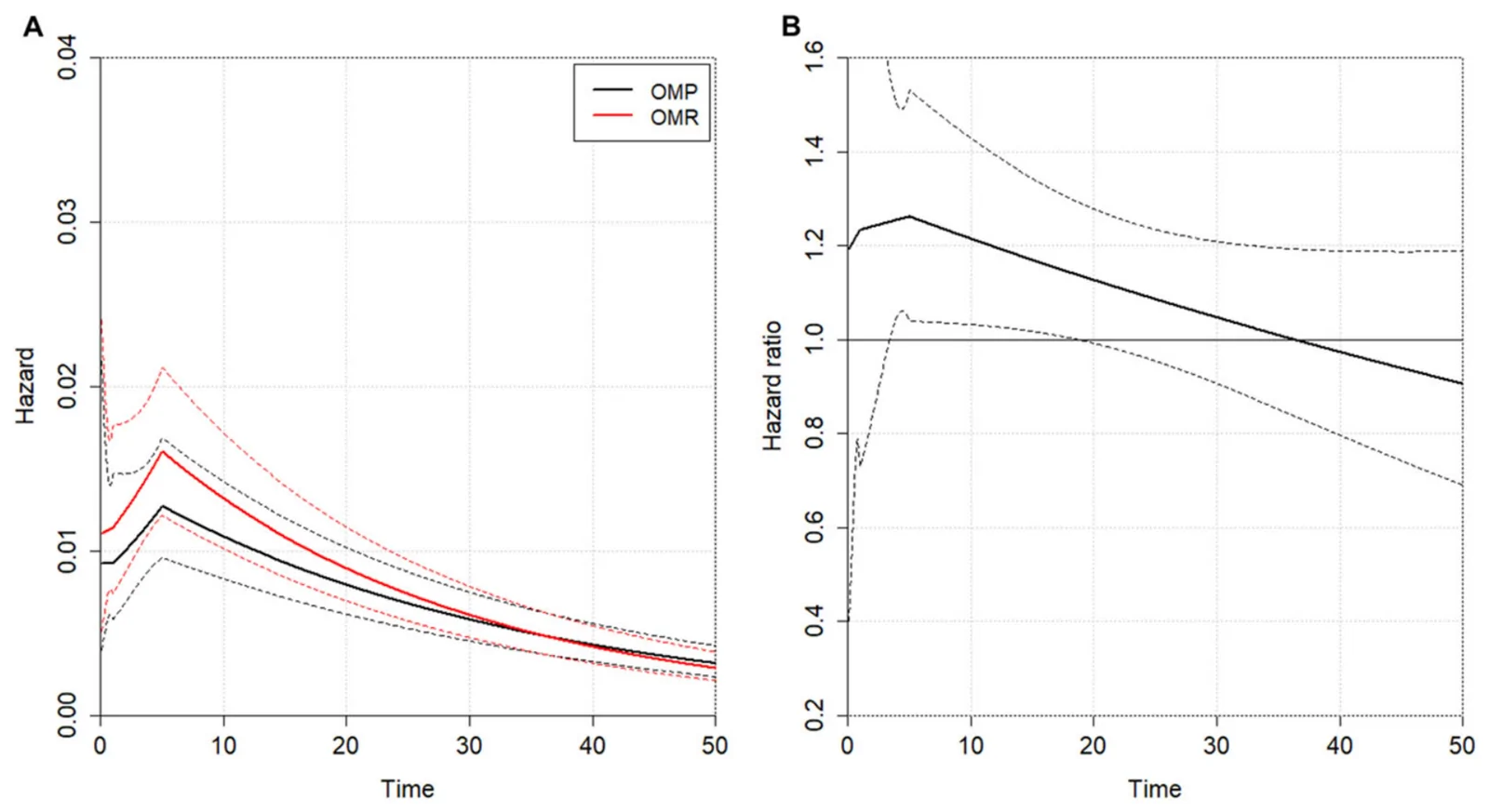
(**A**) Estimated time-dependent DFS hazard functions for OmR (red line) and OmP (black line). (**B**) Estimated time-varying hazard ratio for OmR versus OmP. The solid line represents the predicted disease-free survival curve, whereas the dotted lines denote the corresponding 95% confidence intervals.

The clinical appraisal of the DFS RMSTD was based on studies reporting Kaplan–Meier curves (n = 10). The RMSTD and the time horizons are detailed in Table 3. At the 60-month time horizon, the pooled RMSTD for DFS (OmR minus OmP) was −2.12 months (95% CI, −4.59 to 0.35; *p* = 0.09), with no statistically significant difference in restricted mean disease-free survival between OmR and OmP. For DFS, sensitivity analyses restricted to propensity score-matched cohorts (n = 6 studies; RMSTD −1.99 months, 95% CI −4.33 to 1.56; *p* = 0.18) and studies at moderate risk of bias (n = 5 studies; RMSTD −2.21 months, 95% CI −4.06 to 1.33; *p* = 0.11) yielded results consistent with the primary analysis. The estimated pooled 5-year disease-free survival estimates were 65.0% and 68.1%, respectively (Figure 4). The likelihood-ratio test indicated evidence of a time-varying treatment effect (*p* < 0.001); however, the estimated time-varying hazard ratio did not show a statistically significant difference between OmR and OmP at any point up to 50 months of follow-up (Figure 5A,B).

### 3.5. Secondary Outcomes

OmR was associated with significantly higher intraoperative blood loss (MD 42.9), overall complications (RR 1.17), and longer hospital length of stay (MD 0.49). No statistically significant differences were observed in retrieved lymph nodes, anastomotic leak, operative time, or 30-day mortality (Table 4). Substantial between-study heterogeneity was observed for several perioperative outcomes, particularly blood loss, hospital length of stay, operative time, and lymph node yield; therefore, the magnitude of the corresponding pooled estimates should be interpreted cautiously.

### 3.6. GRADE Evidence

The certainty of evidence according to GRADE was low for overall survival and very low for disease-/relapse-free survival, primarily because of residual confounding and risk of bias inherent to the non-randomized evidence, with additional downgrading for imprecision where applicable (Supplementary Table S3).

## 4. Discussion

This reconstructed IPD meta-analysis provides an updated comparison of oncological and perioperative outcomes following OmR or OmP during gastrectomy for gastric cancer. Routine OmR was not associated with improved OS or DFS compared with OmP. OmR was associated with significantly higher intraoperative blood loss, overall complications, and longer hospital length of stay. No statistically significant differences were observed in retrieved lymph nodes, anastomotic leak, operative time, or 30-day mortality. The present results are consistent with previous comparative studies [31,32,33,45], the majority of which reported no significant differences in long-term survival between the two surgical approaches. By reconstructing individual patient data from published Kaplan–Meier curves, the present analysis extends the available evidence and permits estimation of survival differences in absolute time units rather than relying solely on relative measures of effect. This approach may be particularly informative in surgical oncology, where hazard ratios can be difficult to interpret clinically and may be unreliable when the proportional hazards assumption is not met. The similar pooled 5-year survival estimates are consistent with the absence of an observed survival disadvantage associated with omental preservation.

Gastrectomy with D2 lymphadenectomy remains the standard curative treatment for locally advanced gastric cancer [1,5,46]. Despite radical-intent surgery, recurrence develops in 40–61% of patients and remains the principal cause of treatment failure, with peritoneal dissemination representing the most frequent pattern of relapse [10,47]. Within the peritoneal cavity, the greater omentum is the site most commonly involved by metastatic disease [48]. Consequently, complete omentectomy has traditionally been incorporated into radical gastrectomy based on the hypothesis that the omentum may harbor occult tumor deposits and lymphatic tissue containing disseminated cancer cells. This concept, first proposed by Groves [49], has influenced surgical practice for decades and continues to be reflected in several contemporary guidelines. The Japanese Gastric Cancer Treatment Guidelines recommend complete omentectomy for T3 or more advanced tumors while permitting preservation beyond 3 cm from the gastroepiploic arcade for T1–T2 disease [3], recommendations that have also been adopted by the Italian Research Group for Gastric Cancer (GIRCG) [50]. Similarly, the Chinese Society of Clinical Oncology advocates routine omentectomy during both D1 and D2 gastrectomy [51]. In contrast, the Korean Practice Guidelines and the NCCN Guidelines [2] do not provide specific recommendations regarding omentectomy, highlighting the persistent uncertainty surrounding its oncological value.

The biological rationale for routine OmR reflects the unique anatomical and immunological properties of the greater omentum. Owing to its rich vascular and lymphatic network, the omentum has long been considered a potential conduit for tumor dissemination, particularly in advanced gastric cancers with serosal invasion. Free cancer cells exfoliated from the primary tumor may become trapped within omental milky spots, specialized lymphoid aggregates that are highly vascularized and communicate directly with the peritoneal cavity [52]. Experimental studies have identified these structures as preferential implantation sites for disseminated tumor cells and the earliest foci of peritoneal carcinomatosis [11,12,13,53], providing the historical justification for routine omentectomy. However, accumulating evidence indicates that the greater omentum also functions as an important immunological organ [54]. Milky spots recruit macrophages, neutrophils, natural killer cells, dendritic cells, and lymphocytes, facilitating phagocytosis, antigen presentation, cytokine production, and local activation of innate and adaptive immune responses [55,56]. In addition to supporting angiogenesis and wound healing following abdominal surgery [54], these immune functions may contribute to the clearance of free intraperitoneal tumor cells. Although the clinical relevance of these protective mechanisms remains incompletely understood, they challenge the traditional view of the omentum as merely a passive reservoir for metastatic disease.

The oncological value of routine omentum resection (OmR) remains uncertain as gastric cancer surgery increasingly adopts a more tailored, evidence-based approach. In the present reconstructed individual patient data (IPD) meta-analysis, restricted mean survival time difference (RMSTD) analysis demonstrated no significant difference in 5-year OS between OmR and OmP (−0.79 months, *p* = 0.24). DFS was similarly comparable (RMSTD −2.12 months, *p* = 0.09). These findings are in keeping with previous observational studies. Seo et al. [19] reported similar 5-year OS following propensity score adjustment (72.6 versus 75.4 per cent; *p* = 0.057). Likewise, Huang et al. [26] found no difference in OS in a retrospective cohort of 1615 patients (HR 1.00, 95% c.i. 0.83 to 1.20; *p* = 0.96), and Ri et al. [16] observed comparable 5-year survival in patients with cT3–4 disease (77.1 versus 79.4 per cent; *p* = 0.75). Jagric et al. [25] also found no survival benefit associated with OmR and reported that omentum preservation was not independently associated with an increased risk of peritoneal recurrence. The absence of a survival advantage with routine OmR may reflect the low prevalence of occult omental involvement in patients with resectable gastric cancer without macroscopic disease. Pathological studies have reported omental lymph node metastases or tumor deposits in approximately 2–8 per cent of patients undergoing gastrectomy [57]. Importantly, patients with omental metastases consistently experienced poor long-term outcomes [58], suggesting that omental involvement represents a manifestation of biologically advanced disease rather than an isolated site amenable to surgical clearance [59]. This interpretation is further supported by the frequent association between omental metastases and adverse pathological features, including serosal invasion, linitis plastica, non-curative resection, and other indicators of disseminated tumor biology. Beyond its anatomical role, the greater omentum is increasingly recognized as an immunologically active organ that contributes to peritoneal immune surveillance through resident macrophages, lymphocytes, and other immune cell populations [11,12,13]. Preservation of these functions may theoretically enhance the clearance of free intraperitoneal tumor cells, although the clinical relevance of this mechanism remains to be established. Taken together, these findings question the need for routine omentectomy during curative gastrectomy and suggest that omental preservation may be considered in selected patients, although its oncological safety requires confirmation in prospective randomized trials.

The secondary outcomes provide further information on the perioperative results associated with OmP. OmR was associated with greater intraoperative blood loss, a higher risk of overall postoperative complications, and longer hospital stay, whereas no significant differences were observed for the remaining evaluated outcomes. The similar number of retrieved lymph nodes is particularly relevant, as nodal yield remains an established indicator of surgical quality and accurate pathological staging following D2 gastrectomy. The comparable lymph node harvest is biologically plausible. Anatomical studies have shown that lymph nodes within the greater omentum are concentrated along the gastroepiploic vascular arcade. Howell et al. [60] demonstrated by CT angiography that most omental lymph nodes are located within 7 ± 6 mm of the right gastroepiploic vessels, supporting the concept that complete lymphadenectomy can be achieved without routine resection of the entire greater omentum. This observation is consistent with the Japanese Gastric Cancer Association recommendation that omentectomy extending approximately 4 cm from the gastroepiploic arcade is sufficient during standard gastrectomy [3]. Accordingly, preservation of the distal greater omentum is unlikely to compromise lymphadenectomy or pathological staging when oncological principles are respected. In contrast, OmR was associated with greater intraoperative blood loss, consistent with previous reports [20,21,24,25,26]. Although the absolute increase in blood loss was modest, the higher risk of overall postoperative complications and longer hospital stay suggest that routine omentectomy may increase operative invasiveness without providing measurable oncological benefit.

The present findings should be interpreted in light of several factors that may have influenced the comparison between omentectomy and omentum preservation. First, the increasing use of minimally invasive gastrectomy, including laparoscopic, may have affected omental management [61,62]. Complete omentectomy is technically more demanding in minimally invasive surgery, whereas omentum preservation simplifies dissection from the transverse mesocolon and may therefore have influenced treatment allocation and operative outcomes [20,21,24,25,26]. Second, learning-curve effects related to minimally invasive gastrectomy and omental handling may have improved perioperative outcomes over time, independently of the extent of omental resection [63]. Of the fifteen studies, only one defined the operating surgeon’s proficiency in performing open and laparoscopic gastrectomy. Differences in lymphadenectomy also warrant consideration [64]. Patients underwent D1, D1+, or D2 lymphadenectomy according to tumor stage, patient characteristics, operating surgeon preferences, and contemporary recommendations; as nodal dissection influences operative complexity, morbidity, and staging accuracy, it may have affected both short- and long-term outcomes independently of omental management. Moreover, the broader adoption of D2 lymphadenectomy over time may have coincided with changes in the use of omentectomy or omentum preservation. The widespread implementation of neoadjuvant, perioperative systemic therapy, and focused immunotherapy has altered the management of resectable gastric cancer [65,66,67,68,69,70,71,72,73,74]. By modifying tumor biology and improving long-term outcomes, systemic therapy may have attenuated differences attributable to the surgical approach, while evolving treatment strategies may also have influenced patient selection. Finally, postoperative complications may negatively influence long-term survival, as supported by recent evidence [75,76,77]. These considerations underscore the need for caution when interpreting the present findings and highlight the importance of integrating complication-related outcomes into the overall assessment of therapeutic effectiveness.

This study has several strengths. First, it represents an updated synthesis of the available comparative literature and includes a large overall study population with gastric cancer. Second, the use of reconstructed individual patient data allowed a more refined analysis of time-to-event outcomes than conventional aggregate-data meta-analysis. Third, the application of restricted mean survival time difference provides an absolute estimate of survival gain or loss, facilitating clinical interpretation. Finally, the use of flexible hazard-based modelling enabled formal consideration of time-varying treatment effects [39,40,41,42,43], which is particularly important given the evidence of non-proportional hazards in the present dataset. Several limitations should nevertheless be acknowledged. Most included studies were retrospective cohort studies, and although several used propensity score matching or multivariable adjustment, residual confounding cannot be excluded. Treatment allocation may have been influenced by surgeon preference, institutional protocols, tumor location, perceived disease extent, surgical approach, or period-specific changes in perioperative management. In addition, heterogeneity existed across studies with regard to geographic setting, surgical technique, extent of lymphadenectomy, use of minimally invasive surgery, and systemic therapy protocols. These factors may limit the generalizability of pooled estimates. Reconstructed individual patient data were derived from published Kaplan–Meier curves rather than original patient-level datasets and therefore could not account for unreported covariates or details of neoadjuvant and adjuvant chemotherapy. Furthermore, the definitions of DFS and RFS were not consistently reported across the included studies, precluding a standardized classification of survival events and potentially introducing additional outcome heterogeneity. Stage-stratified survival data were reported in only 8 studies, precluding meaningful stage-specific analyses. Moreover, aggregate published data did not allow reconstruction of recurrence patterns, including the specific site and type of recurrence. Partial omentectomy and distal omental preservation were pooled within the same comparator group, according to the definitions adopted in the original studies. A formal subgroup analysis by preservation technique was not feasible because of inconsistent reporting of operative details across studies. Finally, the lack of dedicated subgroup analyses according to tumor histology, particularly cohesive versus non-cohesive subtypes, and genetic characterization represents an additional potential source of residual confounding [78,79,80].

## 5. Conclusions

This systematic review and reconstructed individual patient data meta-analysis found no significant differences in overall or disease-free survival between omentectomy and omental preservation during curative gastrectomy for gastric cancer. Omentectomy appeared to be associated with greater blood loss, more postoperative complications, and longer hospital stay, without demonstrable perioperative benefit. Although these findings challenge the need for routine omentectomy, the predominantly observational evidence and low/very low certainty of evidence preclude definitive conclusions. Omental preservation may be an option in selected patients, but randomized trials are needed to establish its oncological safety.

## Figures and Tables

**Table 1:** General characteristics of the included studies. Abbreviations: OmR, omental resection; OmP, omental preservation; PSM, propensity score matching; M, male; F, female; Di, differentiated; Un, undifferentiated; Oth, other histological types; pTNM, pathological tumor–node–metastasis classification; AJCC, American Joint Committee on Cancer; UICC, Union for International Cancer Control; Open, open surgical approach; Lap, laparoscopic surgical approach; Total, total gastrectomy; Distal, distal gastrectomy; nr, not reported.

Author, Country, Year	Study Design	Treatment	Patients n°	Age	Gender M/F	Di/Un/oth	pTNM Classification	Stage I	Stage II	Stage III	Stage IV	Open/Lap	Total/Distal
Kim JH [14], Republic of Korea, 2009 2000–2002	Retrospective	OmR	122	60.7	91/31	41/81/nr	nr	nr	nr	nr	nr	122/0	122/0
		OmP	52	62.4	34/18	17/35/nr		nr	nr	nr	nr	52/0	52/0
Hasegawa [15], Japan, 2012 2000–2009	Retrospective PSM	OmR	98	69	72/26	61/37/nr	UICC TNM 7th Edition	16	40	42	0	98/0	46/52
		OmP	98	68.7	72/26	58/40/nr		21	36	41	0	84/14	37/61
Ri [16], Japan, 2020 2006–2012	Retrospective PSM	OmR	263	66.7	176/87	97/127/39	UICC TNM 8th Edition	28	101	129	5	263/0	107/156
		OmP	263	65.7	181/82	92/135/36		29	96	131	7	263/0	112/151
Sakimura [17], Japan, 2020 2008–2017	Retrospective PSM	OmR	70	65	46/24	24/46/0	UICC TNM 8th Edition	nr	nr	nr	nr	41/29	25/nr
		OmP	70	66.5	48/22	36/33/0		nr	nr	nr	nr	25/45	26/nr
Young [18], USA 2020 2008–2016	Retrospective	OmR	90	69.5	62/28	nr/34/nr	nr	nr	nr	nr	nr	nr	25/31
		OmP	381	68	217/164	nr/118/nr		nr	nr	nr	nr	nr	99/140
Seo [19], Republic of Korea, 2021 2003–2015	Retrospective PSM	OmR	225	59	131/94	65/160/nr	nr	nr	95	130	nr	69/156	58/167
		OmP	225	56	137/88	68/157/nr		nr	99	126	nr	60/165	56/169
Lee S. [20], Republic of Korea, 2022 2014–2018	Retrospective PSM	OmR	174	59.9	122/52	100/70/4	AJCC TNM 8th Edition	10	43	121	nr	nr	73/101
		OmP	248	61.6	177/71	130/115/3		21	77	150	nr	nr	91/157
Nonaka [21], Japan, 2022 2002–2016	Retrospective PSM	OmR	23	69	16/7	8/15/0	UICC TNM 8th Edition	0	17	6	0	nr	7/16
		OmP	23	69	13/10	8/15/0		0	16	7	0	nr	5/18
Song [22], China, 2023 2019–2022	Retrospective	OmR	50	nr	29/21	24/26/0	nr	nr	nr	nr	nr	0/50	0/50
		OmP	58	nr	29/29	29/29/0		nr	nr	nr	nr	0/58	0/58
Lee H. [23], Republic of Korea, 2023 2009–2016	Retrospective PSM	OmR	107	65	70/37	36/71/0	nr	nr	nr	nr	nr	0/107	0/107
		OmP	107	62	70/37	36/71/0		nr	nr	nr	nr	0/107	0/107
Jeong [24], Republic of Korea, 2023 2005–2017	Retrospective PSM	OmR	310	58.9	198/112	64/246/nr	AJCC/UJCC 8th Edition	nr	nr	nr	nr	254/56	168/142
		OmP	93	55.9	38/55	17/76/nr		nr	nr	nr	nr	2/91	32/61
Jagric [25], Slovenia, 2024 2015–2022	Retrospective PSM	OmR	98	66.0	70/25	nr	UICC TNM (edition not reported)	35	23	31	3	98/0	64/31
		OmP	100	69	64/36	nr		39	22	33	2	0/98	54/44
Huang [26], Sweden, 2025 2006–2022	Retrospective	OmR	1098	71	627/471	381/182/535	nr	nr	nr	nr	nr	785/110	555/543
		OmP	517	72	305/212	198/110/209		nr	nr	nr	nr	243/195	157/360
Deng [27], China, 2025 2010–2021	Retrospective	OmR	87	52.78	39/48	20/67/0	nr	42	16	35	0	17/70	0/87
		OmP	87	54.40	46/41	22/65/0		36	16	35	0	31/56	0/87
Kocaaslan [28], Turkey, 2026 2010–2023	Retrospective	OmR	61	64	38/23	nr	8th	13	14	34	0	0/61	27/34
		OmP	127	64	84/43	nr		20	31	76	0	0/127	61/66

**Table 2:** Overall survival. Restricted mean survival time differences (RMSTDs) for OmR versus OmP at prespecified time horizons up to 60 months. CI, confidence interval; mos, months.

Time Horizon	No. Trials	RMSTD (mos)	95% CI	*p* Value
12-month	14	−0.01	−0.03, 0.02	0.56
24-month	14	−0.12	−0.38, 0.13	0.35
36-month	14	−0.24	−0.84, 0.34	0.41
48-month	13	−0.56	−1.39, 0.26	0.18
60-month	8	−0.79	−2.13, 0.54	0.24

**Table 3:** Disease Free survival. Restricted mean survival time differences (RMSTDs) for OmR versus OmP at prespecified time horizons up to 60 months. CI, confidence interval; mos, months.

Time Horizon	No. Trials	RMSTD (mos)	95% CI	*p* Value
12-month	10	−0.14	−0.39, 0.10	0.24
24-month	10	−0.76	−1.57, 0.04	0.06
36-month	10	−1.43	−2.81, 0.05	0.06
48-month	9	−2.03	−3.96, 0.11	0.07
60-month	7	−2.12	−4.59, 0.35	0.09

**Table 4:** Secondary outcomes for the comparison OmR vs. OmP. Effect estimates are expressed as risk ratios (RRs) for dichotomous outcomes and mean differences (MDs) for continuous outcomes, with corresponding 95% confidence intervals. I^2^, heterogeneity.

Secondary Outcomes	No. Trials	No. pts	Effect Estimate (95% CI)	I^2^ (%)
Intestinal obstruction	6	1532	1.13 [0.47; 2.70]	0.0
HLOS (days)	8	2395	0.49 [0.06; 0.91]	96.2
30-day mortality	5	3227	0.87 [0.45; 1.67]	0.0
Blood loss (ml)	10	3939	42.9 [7.88; 78.04]	99
Harvested lymph nodes	11	4540	0.02 [−1.41; 1.44]	89.8
Operative time (minutes)	12	4608	12.2 [−5.82; 30.19]	98.8
Anastomotic leak	7	3886	1.16 [0.81; 1.67]	0.0
Intra-abdominal abscess	5	1726	1.65 [0.67; 4.06]	44.5
Postoperative complications	13	4676	1.17 [1.05; 1.36]	26.6

## Data Availability

The data collected and analyzed during the current review are available from the corresponding author on reasonable request.

## Supplementary Materials

The following supporting information can be downloaded at: https://www.mdpi.com/article/10.3390/jcm15218495/s1, Supplementary Figure S1. Restricted mean survival time difference (RMSTD) for OS. Each color represents a single study. The pooled RMSTD with relative 95% CI using different statistical methods estimation is represented by black points (triangle, circle). Supplementary Figure S2. Restricted mean survival time difference (RMSTD) for DFS. Each color represents a single study. The pooled RMSTD with relative 95% CI using different statistical methods estimation is represented by black points (triangle, circle). Supplementary Table S1. Definition of OmP according to the included studies. Supplementary Table S2. Risk of bias assessment of included non-randomized studies according to ROBINS-I. Abbreviations: ROBINS-I, Risk Of Bias In Non-randomized Studies of Interventions; D1, bias due to confounding; D2, bias in selection of participants into the study; D3, bias in classification of interventions; D4, bias due to deviations from intended interventions; D5, bias due to missing data; D6, bias in measurement of outcomes; D7, bias in selection of the reported result. Supplementary Table S3: GRADE evaluation. Legend. (a). Risk of bias: Risk of bias was downgraded by two levels because ROBINS-I identified substantial concerns among the studies contributing to the oncological outcomes, primarily related to residual confounding in treatment allocation, including tumor extent, surgical approach, institutional practice, treatment period, and surgeon preference. (b). Inconsistency: downgraded by one level when substantial between-study heterogeneity was present (I^2^ approximately 90% or greater). (c). Indirectness: not downgraded because the populations, interventions, comparators, and outcomes were considered sufficiently relevant to the review question, despite clinical heterogeneity in stage, operative approach, and perioperative management. (d). Imprecision: downgraded when confidence intervals crossed the null and included potentially important benefit and harm; downgraded by two levels for particularly wide confidence intervals. (e). Publication bias: no downgrade was applied because there was no clear evidence of publication bias; however, formal assessment was limited for outcomes informed by a small number of studies. (f). Overall survival: although the 95% CI crossed the null, the absolute RMSTD interval was relatively narrow and did not support a clinically meaningful survival advantage for routine omentectomy; therefore, no additional downgrade for imprecision was applied. (g). Disease-free survival: downgraded by one level for imprecision because the 60-month confidence interval included no effect and an absolute difference of several months in favor of omental preservation. Table S4. PRISMA checklist.

## Abbreviations

The following abbreviations are used in this manuscript:

ASA	American Society of Anesthesiologists
BMI	Body mass index
CI	Confidence interval
CSS	Cancer-specific survival
DFS	Disease-free survival
ESMO	European Society for Medical Oncology
GC	Gastric cancer
HRs	Hazard ratios
IPD	Individual patient data
MD	Mean difference
OmP	Omental preservation
OmR	Omental resection
ORs	Odds ratios
OS	Overall survival
RMSTD	Restricted mean survival time difference
SMD	Standardized mean difference

## Appendix A. Search Strategy

**PubMed/MEDLINE**
("Omentum"[MeSH Terms] OR "omentectomy"[Title/Abstract] OR "omental resection"[Title/Abstract] OR "greater omentectomy"[Title/Abstract] OR "total omentectomy"[Title/Abstract] OR "partial omentectomy"[Title/Abstract] OR "oment* preserv*"[Title/Abstract])
AND
("Gastrectomy"[MeSH Terms] OR "Stomach Neoplasms"[MeSH Terms] OR "gastrectomy"[Title/Abstract] OR "gastric cancer"[Title/Abstract] OR "gastric carcinoma"[Title/Abstract] OR "stomach cancer"[Title/Abstract] OR "stomach neoplasm*"[Title/Abstract])
AND
("Survival"[MeSH Terms] OR "Survival Analysis"[MeSH Terms] OR "overall survival"[Title/Abstract] OR "disease-free survival"[Title/Abstract] OR "recurrence-free survival"[Title/Abstract] OR "cancer-specific survival"[Title/Abstract] OR "cancer specific survival"[Title/Abstract] OR "survival"[Title/Abstract])
**Scopus**
TITLE-ABS-KEY(
(omentectomy OR "omental resection" OR "greater omentectomy" OR "total omentectomy" OR "partial omentectomy" OR "omentum preservation" OR "omental preservation")
AND
(gastrectomy OR "gastric cancer" OR "gastric carcinoma" OR "stomach cancer" OR "stomach neoplasm*")AND(survival OR "overall survival" OR "disease-free survival" OR "recurrence-free survival" OR "cancer-specific survival" OR "cancer specific survival"))
Web of Science
TS=(
(omentectomy OR "omental resection" OR "greater omentectomy" OR "total omentectomy" OR "partial omentectomy" OR "omentum preservation" OR "omental preservation")
AND
(gastrectomy OR "gastric cancer" OR "gastric carcinoma" OR "stomach cancer" OR "stomach neoplasm*")
AND
(survival OR "overall survival" OR "disease-free survival" OR "recurrence-free survival" OR "cancer-specific survival" OR "cancer specific survival")
)
Cochrane Central Register of Controlled Trials (CENTRAL)
([mh Omentum] OR omentectomy OR "omental resection" OR "greater omentectomy" OR "total omentectomy" OR "partial omentectomy" OR "omentum preservation" OR "omental preservation")
AND
([mh Gastrectomy] OR [mh "Stomach Neoplasms"] OR gastrectomy OR "gastric cancer" OR "gastric carcinoma" OR "stomach cancer")
AND
([mh Survival] OR [mh "Survival Analysis"] OR survival OR "overall survival" OR "disease-free survival" OR "recurrence-free survival" OR "cancer-specific survival" OR "cancer specific survival")
Google Scholar
("omentectomy" OR "omental resection" OR "omentum preservation") AND ("gastrectomy" OR "gastric cancer") AND ("survival" OR "overall survival" OR "disease-free survival" OR "cancer-specific survival")
