# Supplementary material for: Omentectomy Versus Omental Preservation During Gastrectomy for Gastric Cancer: Systematic Review and Reconstructed Individual Patient Data Meta-Analysis
Source: J Clin Med. 2026 Nov 1;15(21):8495. doi: 10.3390/jcm15218495 (PMC13764339; doi:10.3390/jcm15218495)
Supplement: Supplementary file 1 [file jcm-15-08495-s001.zip › Supplementary Table S1 .pdf]

Supplementary Table S1. Surgical definitions of greater omentum preservation across included studies

**Study**	**Dissection distance**	**Operative description**
**Ji/Kim 2009**	±4 cm	The greater omentum was divided at least approximately 4 cm from the gastroepiploic vessels.
**Hasegawa 2012**	±3 cm	Division was performed 3 cm from the gastroepiploic arcade, preserving the colonic portion of the greater omentum.
**Ri 2020**	≥3 cm	The greater omentum was divided at least 3 cm from the gastroepiploic artery; the remaining omentum was left attached to the colon.
**Sakimura 2020**	≥3 cm	Dissection was generally performed at least 3 cm below the greater curvature, along the gastroepiploic vessels; when the stomach–colon distance was short, the sparse omental area was dissected.
**Young 2020**	NR	Operative definition not reported.
**Seo 2021**	3–4 cm	The omentum was divided 3–4 cm from the gastroepiploic arcade, preserving the remaining portion.
**Lee S. 2022**	≈4 cm	The greater omentum was resected within approximately 4 cm of the gastroepiploic vascular arcade, preserving the remainder.
**Nonaka 2022**	3–4 cm	During radical gastrectomy, the omentum was divided 3–4 cm from the gastroepiploic arcade.
**Song 2023**	±3 cm	The greater omentum was divided 3 cm from the gastroepiploic vascular arcade, preserving its colonic portion.
**Lee H. 2023**	3–4 cm	Division began 4–5 cm from the gastroepiploic arcade using ultrasonic shears and proceeded toward the origin of the left gastroepiploic vessels.
**Jeong 2024**	Not specified	The extent of omentectomy was determined by surgeon preference and patient characteristics, including comorbidities and performance status.
**Jagric 2024**	±3 cm	The gastrocolic ligament was incised beginning 3 cm from the gastroepiploic arcade.
**Huang 2025**	≥3 cm	The greater omentum was typically divided at least 3 cm below the right and left gastroepiploic arteries, along the cranial border of the transverse colon.
**Deng 2025**	±3 cm	The greater omentum was divided more than 3 cm from the gastroepiploic vascular arcade, preserving the colonic portion.
**Kocaaslan 2026**	3–5 cm	Partial omentectomy was performed 3–5 cm distal to the gastroepiploic arcade.

Abbreviations: NR, not reported. Distances refer to the reported dissection line relative to the gastroepiploic vascular arcade unless otherwise specified.
