# Supplementary material for: Omentectomy Versus Omental Preservation During Gastrectomy for Gastric Cancer: Systematic Review and Reconstructed Individual Patient Data Meta-Analysis
Source: J Clin Med. 2026 Nov 1;15(21):8495. doi: 10.3390/jcm15218495 (PMC13764339; doi:10.3390/jcm15218495)
Supplement: Supplementary file 1 [file jcm-15-08495-s001.zip › Supplementary Table S2.pdf]

Study	D1: Confounding	D2: Selection of participants	D3: Classification of interventions	D4: Deviations from intended interventions	D5: Missing data	D6: Measurement of outcomes	D7: Selection of reported result	Overall risk of bias
**Ji/Kim 2009**	Serious	Moderate	Low	Low	Moderate	Moderate	Moderate	**SERIOUS**
**Hasegawa 2012**	Serious	Moderate	Low	Moderate	Moderate	Moderate	Moderate	**SERIOUS**
**Ri 2020**	Moderate	Low	Low	Low	Moderate	Moderate	Low	**MODERATE**
**Sakimura 2020**	Moderate	Moderate	Low	Low	Low	Moderate	Moderate	**MODERATE**
**Young 2020**	Serious	Low	Low	Low	Moderate	Low	Moderate	**SERIOUS**
**Seo 2021**	Moderate	Moderate	Low	Low	Moderate	Moderate	Moderate	**MODERATE**
**Lee S. 2022**	Moderate	Moderate	Low	Low	Low	Moderate	Moderate	**MODERATE**
**Nonaka 2022**	Moderate	Moderate	Low	Low	Moderate	Moderate	Moderate	**MODERATE**
**Song 2023**	Serious	Moderate	Low	Low	Moderate	Moderate	Moderate	**SERIOUS**
**Lee H. 2023**	Serious	Moderate	Low	Low	Moderate	Moderate	Moderate	**SERIOUS**
**Jeong 2024**	Serious	Moderate	Low	Low	Moderate	Moderate	Moderate	**SERIOUS**
**Jagric 2024**	Serious	Serious	Low	Low	Low	Moderate	Moderate	**SERIOUS**
**Huang 2025**	Moderate	Moderate	Low	Low	Low	Low	Moderate	**MODERATE**
**Deng 2025**	Serious	Moderate	Low	Low	Low	Low	Moderate	**SERIOUS**
**Kocaaslan 2026**	Serious	Serious	Low	Low	Serious	Moderate	Moderate	**SERIOUS**

Supplementary Table S2. Risk of bias assessment of included non-randomized studies according to ROBINS-I. Abbreviations: ROBINS-I, Risk Of Bias In Non-randomized Studies of Interventions; D1, bias due to confounding; D2, bias in selection of participants into the study; D3, bias in classification of interventions; D4, bias due to deviations from intended interventions; D5, bias due to missing data; D6, bias in measurement of outcomes; D7, bias in selection of the reported result;
