# Supplementary material for: Omentectomy Versus Omental Preservation During Gastrectomy for Gastric Cancer: Systematic Review and Reconstructed Individual Patient Data Meta-Analysis
Source: J Clin Med. 2026 Nov 1;15(21):8495. doi: 10.3390/jcm15218495 (PMC13764339; doi:10.3390/jcm15218495)
Supplement: Supplementary file 1 [file jcm-15-08495-s001.zip › Supplementary Table S3.pdf]

Outcome	Studies / participants	Effect estimate	Risk of bias	Inconsistency	Indirectness	Imprecision	Publication bias	Certainty
**Overall survival, 60-month**	8 studies	RMSTD −0.79 mo (95% CI −2.13 to 0.54)	Very serious −2	Not serious	Not serious	Not serious*	Not suspected	**LOW ⊕⊕◯◯**
**Disease-/relapse-free survival, 60-month**	6 studies	RMSTD −2.12 mo (95% CI −4.59 to 0.35)	Very serious −2	Not serious	Not serious	Serious −1	Not suspected	**VERY LOW ⊕◯◯◯**

Supplementary Table S3: GRADE evaluation. Legend.

a. Risk of bias: Risk of bias was downgraded by two levels because ROBINS-I identified substantial concerns among the studies contributing to the oncological outcomes, primarily related to residual confounding in treatment allocation, including tumor extent, surgical approach, institutional practice, treatment period, and surgeon preference.
b. Inconsistency: downgraded by one level when substantial between-study heterogeneity was present (I^2 approximately 90% or greater).
c. Indirectness: not downgraded because the populations, interventions, comparators, and outcomes were considered sufficiently relevant to the review question, despite clinical heterogeneity in stage, operative approach, and perioperative management.
d. Imprecision: downgraded when confidence intervals crossed the null and included potentially important benefit and harm; downgraded by two levels for particularly wide confidence intervals.
e. Publication bias: no downgrade was applied because there was no clear evidence of publication bias; however, formal assessment was limited for outcomes informed by a small number of studies.
f. Overall survival: although the 95% CI crossed the null, the absolute RMSTD interval was relatively narrow and did not support a clinically meaningful survival advantage for routine omentectomy; therefore, no additional downgrade for imprecision was applied.
g. Disease-free survival: downgraded by one level for imprecision because the 60-month confidence interval included no effect and an absolute difference of several months in favor of omental preservation.
